# Direct synthesis of carbon nanofibers from South African coal fly ash

**DOI:** 10.1186/1556-276X-9-387

**Published:** 2014-08-10

**Authors:** Nomso Hintsho, Ahmed Shaikjee, Hilary Masenda, Deena Naidoo, Dave Billing, Paul Franklyn, Shane Durbach

**Affiliations:** 1 DST-NRF Centre of Excellence in Strong Materials, University of the Witwatersrand (Wits), Private Bag 3, Johannesburg 2050, South Africa; 2 Molecular Sciences Institute, School of Chemistry, University of the Witwatersrand (Wits), Private Bag 3, Johannesburg 2050, South Africa; 3 School of Physics, University of the Witwatersrand (Wits), Private Bag 3, Johannesburg 2050, South Africa

**Keywords:** Fly ash, Catalytic chemical vapour decomposition, Carbon nanofibers, Iron

## Abstract

Carbon nanofibers (CNFs), cylindrical nanostructures containing graphene, were synthesized directly from South African fly ash (a waste product formed during the combustion of coal). The CNFs (as well as other carbonaceous materials like carbon nanotubes (CNTs)) were produced by the catalytic chemical vapour deposition method (CCVD) in the presence of acetylene gas at temperatures ranging from 400°C to 700°C. The fly ash and its carbonaceous products were characterized by transmission electron microscopy (TEM), thermogravimetric analysis (TGA), laser Raman spectroscopy and Brunauer-Emmett-Teller (BET) surface area measurements. It was observed that as-received fly ash was capable of producing CNFs in high yield by CCVD, starting at a relatively low temperature of 400°C. Laser Raman spectra and TGA thermograms showed that the carbonaceous products which formed were mostly disordered. Small bundles of CNTs and CNFs observed by TEM and energy-dispersive spectroscopy (EDS) showed that the catalyst most likely responsible for CNF formation was iron in the form of cementite; X-ray diffraction (XRD) and Mössbauer spectroscopy confirmed these findings.

## Background

The synthesis of carbon nanomaterials (CNMs) has received tremendous interest in the last two decades [1-5]. These endeavours have been driven by the need to exploit the unique chemical and physical properties associated with CNMs (e.g. strength [6,7]), as well as the desire to develop synthetic strategies that are cost-effective and non-destructive to the environment [8-10]. The synthesis of well-structured CNMs is known to require three main components: a source of energy, a source of carbon and a template or catalyst [11]. Recent publications have shown that efforts have focused on using lower energy sources (low-temperature synthesis), natural or recyclable carbon reactants and appropriate templates [12-15].

One of the main challenges in the chemical industry has been the development of low-cost, recyclable and effective substrates (catalysts) upon which well-structured CNMs can grow [16-18]. This has prompted interest in several industrial by-products that contain components that are known to actively decompose carbon reagents into CNMs [19-22]. Of interest has been the study of the effect of coal fly ash as a catalyst for carbon nanomaterial growth. Fly ash is typically a by-product of several energy and power generation industries throughout the world, with an estimated 25 million tons produced annually in South Africa [23]. Currently, only a fraction of this material is utilized effectively, with the remainder proving to be environmentally hazardous due to the presence of several toxic elements like mercury, lead, etc. [24-26]. It has been observed that fly ash can be effective at producing carbon nanotubes (CNTs), provided that the reaction conditions are correct (as summarised below) [13,27,28]. This is due mainly to the transition metal contents in certain fly ashes. Generally, fly ash consists of SiO_2_ (*c.a.* 73.6%), Al_2_O_3_ (*c.a.* 18.7%), Fe_2_O_3_ (*c.a.* 1.9%) and TiO_2_ (*c.a.* 1.4%) and can also include trace amounts of CaO, BaO, MgO, MnO, P_2_O_5_ as well as copper and chromium oxides [29]. However, metals such as Fe/Ni, Ni, Co, Mn, Cu, V, Cr, Mo and Pd have been used in the past as catalysts for CNT and carbon nanofiber (CNF) syntheses [30-35], hence the potential of fly ash to be used as a catalyst in this reaction. In this regard, Yasui et al. [28] have used Japanese fly ash, where Fe was added to the ash to enhance its activity. Although CNTs were produced, these were of a very low yield and poor quality. Dunens et al. [36] showed that CNTs and CNFs could be produced by Australian coal fly ash using the chemical vapour deposition (CVD) method. However, in their case, multiple steps were followed, as iron (which was low in their fly ash, <2.5%) also had to be impregnated into their substrate and ethylene (an expensive carbon source) was used. This therefore resulted in the high cost of CNT and CNF production, although a recycled waste material was used as a catalyst. In an effort to improve the aforementioned processes, Salah et al. [27] used carbon-rich Saudi Arabian fly ash to produce CNTs. These tubes were also synthesized through a CVD process, but pre-treatment of the ash to remove unburned carbon was required in order to use the ash as a catalyst.

Reports on the effectiveness of fly ash as a catalyst or template in the synthesis of CNFs are limited [27,28,36]. Moreover, fly ash is either considered as a support for other more active metallic catalyst particles [28,36] or used after extensive synthetic treatment [27]. On the other hand, no work has been done using the South African coal fly ash to make CNFs.

This article reports a simple, direct route for the synthesis of CNFs from South African coal fly ash and acetylene at varying temperatures. Here no pre-treatments or additions of expensive catalysts were required, as the fly ash was used as received.

## Methods

### Synthesis

Waste South African coal fly ash was obtained from the Electricity Supply Commission (ESCOM) Research and Innovation Centre (Rosherville, South Africa) and was used without any chemical pre-treatments or thermal modifications. Carbon deposition was achieved by the catalytic chemical vapour deposition method (CCVD) of acetylene over the waste coal fly ash. In these reactions, the coal fly ash was the catalyst, acetylene the carbon source and hydrogen the carrier gas, to create an optimal reaction environment [37-39]. In each synthesis run, 500 mg of as-received fly ash was uniformly spread in a small quartz boat and placed in the centre of a horizontal furnace. The coal fly ash was then heated at 10°C/min in H_2_ at 100 ml/min to temperatures between 400°C and 700°C in 100°C increments, where upon acetylene gas was introduced into the reaction zone at 100 ml/min for 30 min. After 30 min of reaction time, the flow of acetylene was terminated and the reactor was cooled under H_2_ to ambient temperature. The resultant carbonaceous material was then harvested for characterization.

### Characterization

To identify the metals and their amounts (Table 1) found in the coal fly ash, X-ray fluorescence (XRF) was employed. The morphologies and particle sizes of the as-received and acetylene-treated fly ash were characterized by transmission electron microscopy (TEM) using a FEI Tecnai G2 Spirit electron microscope (FEI Co., Hillsboro, OR, USA) at an accelerating voltage of 120 kV. Energy-dispersive X-ray spectroscopy (EDS) was used to identify the catalyst/s present in the acetylene-treated fly ash. X-ray diffraction (XRD) and Mössbauer spectroscopy were also used to confirm the catalyst responsible for CNF formation. XRD measurements were carried out with the help of a Bruker D2 phaser (Bruker AXS, Karlsruhe, Germany) in Bragg-Brenton geometry with a Lynexe detector using Cu-Kα radiation at 30 kV and 10 mA. The samples were scanned from 10° to 90° theta (*θ*).

**Table 1:** The chemical composition of South African coal fly ash samples obtained by XRF

**Fly ash component**	**Weight (%)**
SiO_2_	55.03
Al_2_O_3_	27.76
Fe_2_O_3_	0.62
FeO	4.99
MnO	0.08
CaO	5.00
MgO	1.43
Na_2_O	0.14
K_2_O	0.90

Particle size distributions were obtained from the TEM micrographs. The particle size distributions of as-received and acetylene-treated coal fly ash (at different temperatures) were also determined using a Malvern particle size analyser (Master Sizer 2000, Malvern Instruments Ltd., Worcestershire, UK). Both these materials were analysed by dispersing them in two different solutions: (1) water and (2) a Dolapix solution (100 ml water:2 ml Dolapix (Zschimmer & Schwarz, Lahnstein, Germany)). Laser Raman spectroscopy was used to ascertain the type of carbonaceous materials that were formed. The thermal stability of the acetylene-treated fly ash products was determined by using a PerkinElmer Pyris 1 thermogravimetric analyser (TGA; PerkinElmer, Waltham, MA, USA). In these measurements, a 10 mg sample was heated to 900°C at a rate of 10°C/min under air (20 ml/min). The specific surface areas of approximately 200 mg of as-received and acetylene-treated fly ash materials (between 400°C and 700°C) were determined using the Brunauer-Emmett-Teller (BET) surface area method by N_2_ adsorption using an ASAP 2000 Micrometrics Tristar surface area and porosity analyser (Micromeritics Instrument Co., Norcross, GA, USA). Both materials were degassed at 150°C for 4 h under nitrogen before testing to remove the moisture. Mössbauer spectroscopy measurements were carried out in transmission mode with a 10 miC ^57^Co(Rh) source. Measurements were performed at room temperature on the as-received and acetylene-treated fly ash samples at 700°C.

## Results and discussion

### Morphological studies

**Figure 1:**
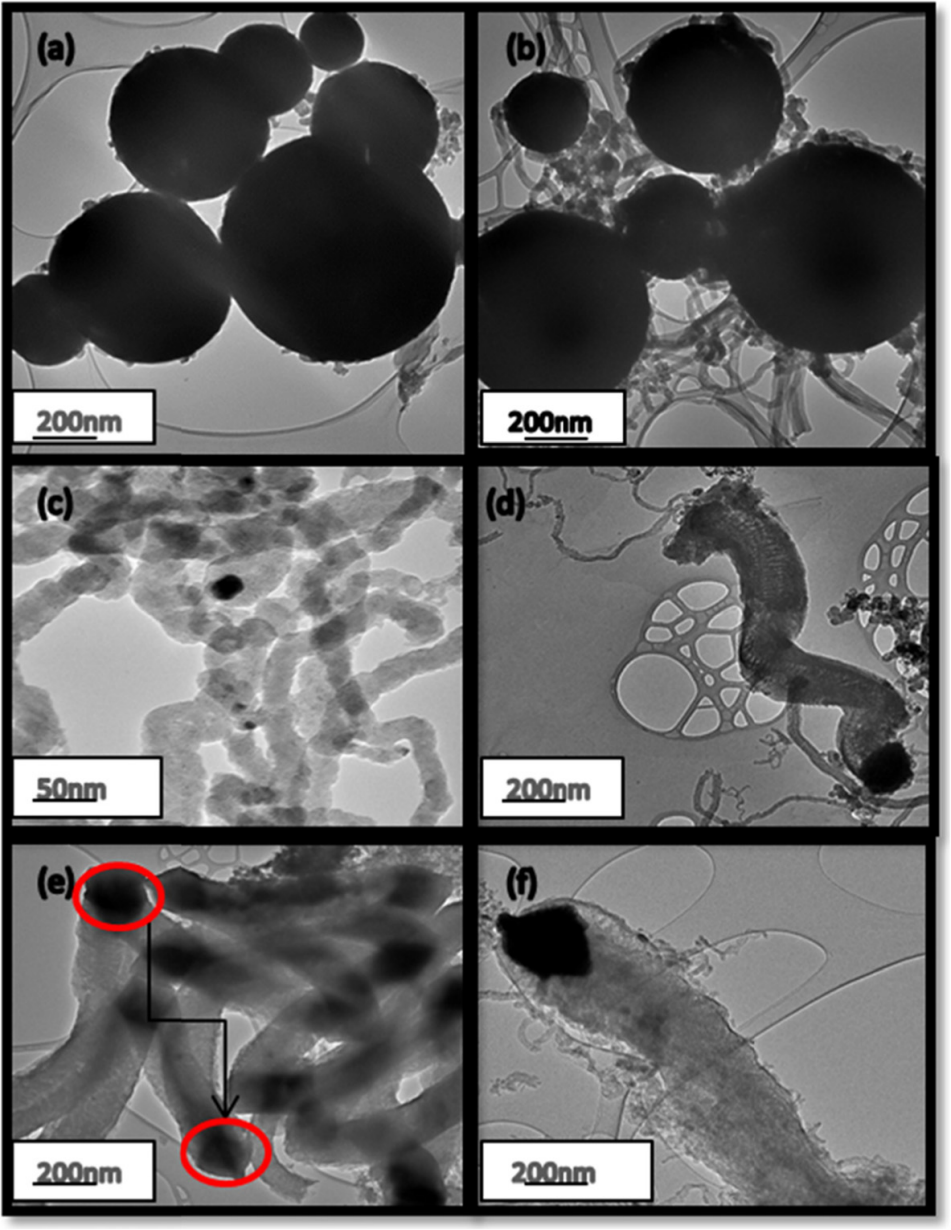
**As-received coal fly ash and synthesised CNFs.** Images of as-received coal fly ash **(a)** and CNFs synthesized at **(b)** 400°C, **(c)** 500°C, **(d)** 600°C and **(e**, **f)** 700°C. In (a), the as-received coal fly ash was observed to be glassy, smooth and spherical in nature. The glassy, smooth-shaped fly ash became covered with regularly and irregularly shaped CNFs. In (c) and (d), large CNFs were intertwined with smaller ones. In (e), well-defined CNFs, apparently formed by tip growth, were clearly visible as seen by the red-coloured circles.

**Figure 2:**
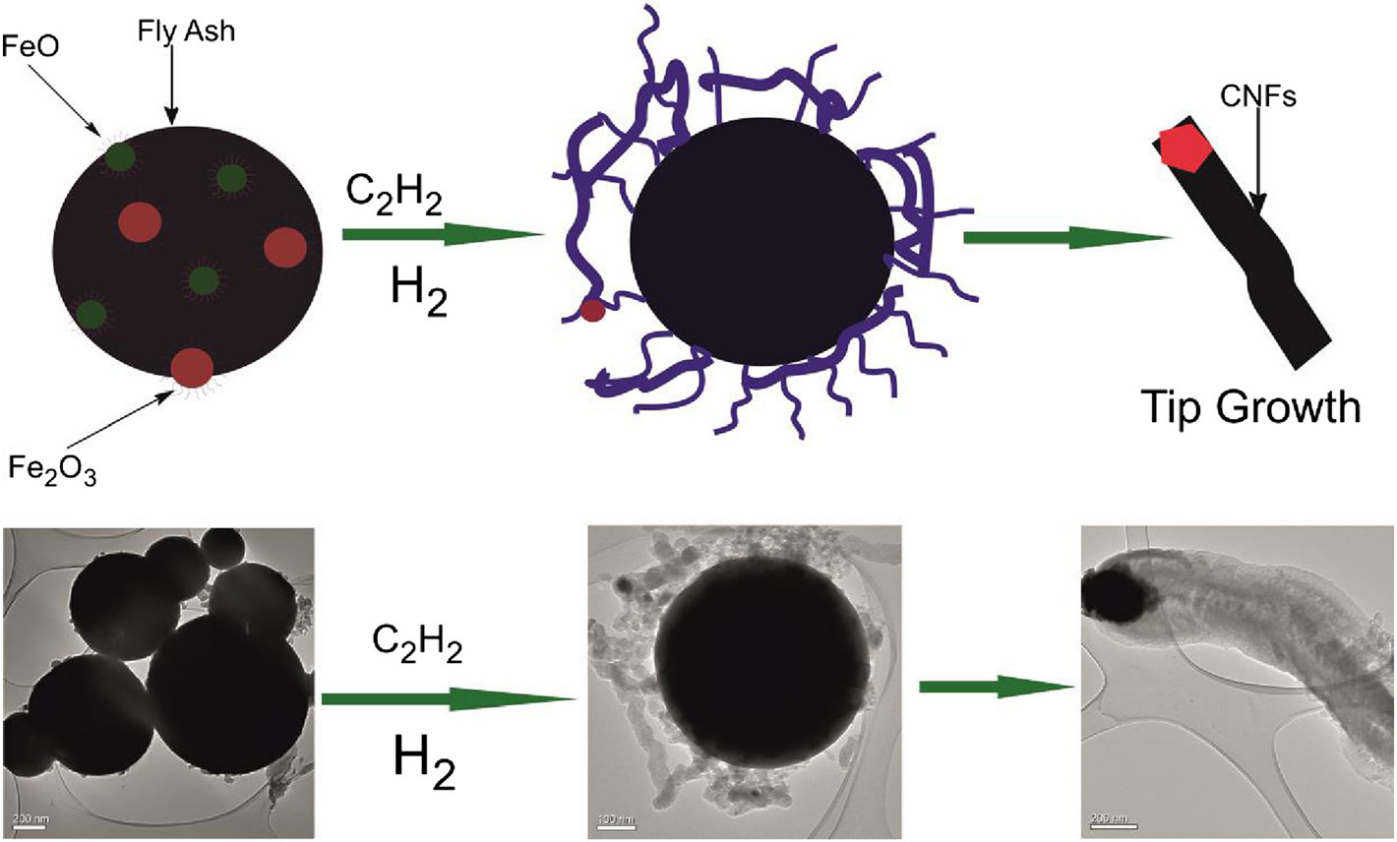
Proposed reaction scheme for CNF growth, using South African coal fly ash as a catalyst.

The sizes, shapes and morphologies of the as-received and acetylene-treated fly ash were investigated using TEM. The results can be observed in Figure 1a,b,c,d,e,f. The as-received fly ash materials (Figure 1a) appeared to be spherically shaped. Fly ash agglomerates shaped like these have often been observed with inorganic salts and may be caused by inter-particulate fusion during the cooling of the fly ash [40]. In Figure 1b,c,d,e, it was observed that the glassy, smooth-shaped fly ash particles began to be coated with regularly and irregularly shaped CNFs when subjected to acetylene. In Figure 1c,d, it was noted that the types of CNMs that were formed varied from large CNFs to smaller CNTs. While the exact growth mechanism of CNTs/CNFs formed from fly ash as a catalyst has not been fully ascertained, it appeared that tip growth could not be discounted (as seen by the red-coloured circles in Figure 1e,f). This type of growth has typically been observed when either iron (Fe) or cobalt (Co) was used as a catalyst for CNM formation. While it is known from previous studies that at least 2.5% of iron is required as a catalyst for CNF formation when using fly ash [36], the XRF data (Table 1) obtained for the South African coal fly revealed that at least 5.6% of iron (in the forms of Fe_2_O_3_ and FeO) was present. Based upon this information and observations made from this research, the reaction scheme in Figure 2 has been proposed.

For this type of growth to occur, it is known that there is normally a weak interaction between the catalyst and support [41]. During this process, the carbon reagent decomposes on the metal particle under specific reaction conditions. The carbon deposited on the metal then either dissolves/re-precipitates to form either CNT/CNFs, or the carbon migrates over the metal particle to form a tube/fibre [41]. If the catalyst particles are large, then multi-walled carbon nanotubes (MWCNTs) and CNFs may be formed [41].

**Figure 3:**
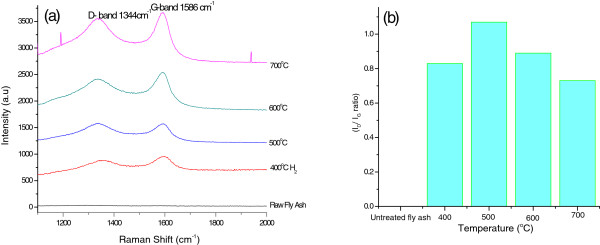
**Raman spectra and *I***_**D**_**/*I***_**G **_**ratios. (a)** Laser Raman spectra of as-received coal fly ash and the products from fly ash exposed to acetylene at various temperatures. **(b)***I*_D_/*I*_G_ ratios of the CNFs synthesized in acetylene. The D and G band peaks confirmed the formation of CNFs that were identified by TEM. CNFs at 500°C displayed the highest degree of disorder.

**Figure 4:**
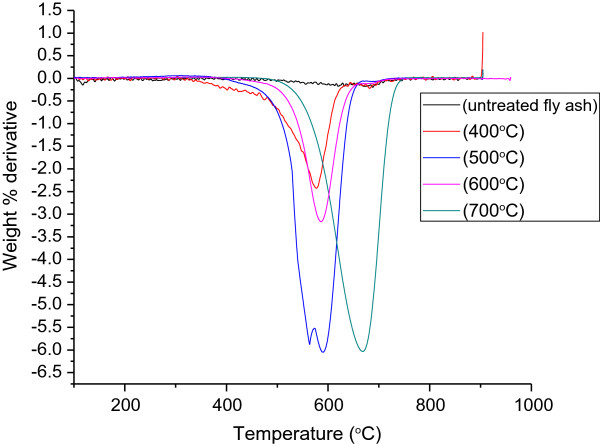
**The first-order weight derivatives of as-received and acetylene-treated coal fly ash at varying temperatures.** CNFs at 700°C displayed the highest oxidation temperature, but CNFs at 500°C displayed a bimodal oxidation profile.

To determine the graphitic nature of the carbonaceous products, laser Raman spectroscopy was conducted. Figure 3 shows the laser Raman spectra that were used to determine the structural information of CNFs produced by the exposure of coal fly ash to acetylene. As expected, the spectrum of the as-received fly ash did not show any peaks, but in the fly ash exposed to acetylene, peaks at 1,350 and 1,590 cm^−1^ were observed. The intensity ratio of these peaks, known as the D band (due to disordered carbon features) and G band (due to the ordered graphitic carbon features), respectively, represents the degree of graphitization of carbon in the reaction products [36]. A low intensity ratio (*I*_D_/*I*_G_) indicates a greater degree of wall graphitization, leading to a superior quality of CNFs and/or CNTs. The intensity ratios of the D and G bands (*I*_D_/*I*_G_) are depicted in Figure 3b. The *I*_D_/*I*_G_ ratio was found to be low at 400°C, indicating that the products contained more graphitic carbon than non-graphitic (non-crystalline) carbon. However, when the reaction temperature was increased to 500°C, the *I*_D_/*I*_G_ ratio was observed to have increased to 1.1 (to the highest value observed in these studies). The results of the TGA analyses (Figure 4) of the carbonaceous products formed at 500°C revealed the presence of two combustion peaks, i.e. two separate CNM products. While the exact reason for the formation of two types of CNMs at this temperature is not fully known, it is believed that this observation most likely accounts for the anomalous increase in the *I*_D_/*I*_G_ ratio. Thereafter, when the reaction temperature was increased to 600°C and 700°C, the *I*_D_/*I*_G_ ratio decreased. This indicated that the degree of disordered carbon that was formed decreased as the temperature was increased. These results showed that the CNFs produced at 700°C had the highest quantity of graphitic carbon and were similar to those reported in previous studies where Fe-supported catalysts were used [42].

### Thermogravimetric studies

Thermogravimetric analyses were carried out to investigate the thermal degradation behaviour of as-received and acetylene-treated fly ash. It has been reported that the graphitic nature of CNMs is directly proportional to their thermal stability [43]. Hence, the first-order weight derivatives of the data so obtained typically gives an indication of the type of carbon present (Figure 4). Typically, highly crystalline nanofibers have been found to be resistant to oxidation when compared to other forms of carbon [44]. Additionally, the diameters and the amount of defects in such materials have also been known to influence their oxidation temperatures [36]. From the TGA thermograms, it was observed that all of the CNMs produced had final oxidation temperatures that were greater than 550°C. However, as previously stated, at least two different forms of carbon were synthesized when the reaction temperature was 500°C. These may have arisen due to the poor carbonization of acetylene, leading to impurities such as amorphous carbon and hence the formation of a higher degree of non-graphitic carbonaceous materials, as confirmed by the laser Raman results (Figure 3a). However, CNFs synthesized at 700°C had the highest oxidation temperature (*c.a.* 690°C). These results concurred with the laser Raman data, where CNFs formed at 700°C displayed the lowest *I*_D_/*I*_G_ ratio, i.e. they were the most graphitic.

### Particle size and surface area measurements

**Figure 5:**
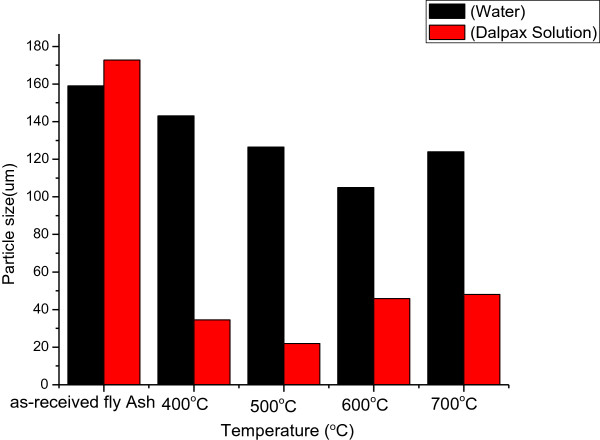
Varying particle sizes of the coal fly ash samples exposed to acetylene at different temperatures.

**Figure 6:**
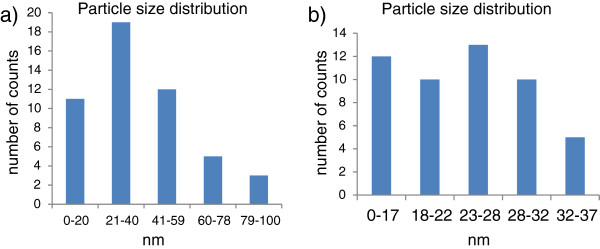
**Particle size distribution. (a)** As-received coal fly ash. **(b)** Acetylene-treated coal fly ash at 500°C.

**Figure 7:**
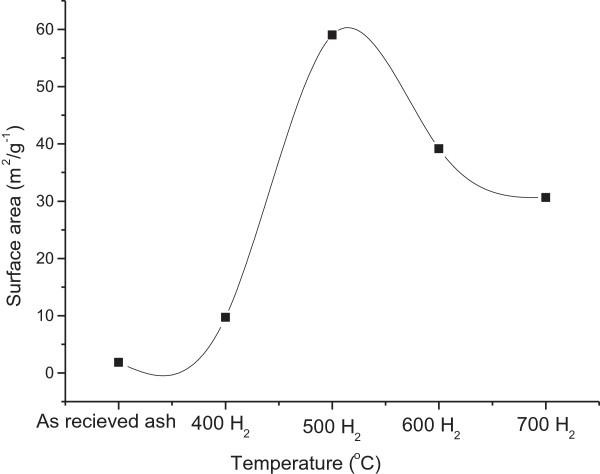
**BET surface areas.** BET surface areas of CNFs synthesized by exposure of coal fly ash to acetylene at temperatures from 400°C to 700°C in H_2**.**_ The CNFs formed at 500°C had the highest surface area, which corresponded to the lowest particle size.

The particle sizes and surface areas of the as-received and acetylene-treated coal fly ash which reacted at temperatures between 400°C and 700°C are depicted in Figures 5,6,7. As-received coal fly ash, when analysed in water, had a particle size of 160 μm. After exposure to acetylene at 700°C, this size was reduced to 130 μm. A small reduction in the particle size was anticipated, as the fly ash particles were entrained in the CNFs, hence reducing their agglomeration. Likewise, although the CNFs are known to be hydrophobic and not easily dispersed in water [45], the entrained fly ash most probably enhanced their solubility. Both of these materials were then introduced into a Dolapix polymer solution. Dolapix solution is known to have the ability to disperse such materials evenly, reducing cluster formation and agglomeration [46]. However, in the Dolapix solution, the particle size for the as-received coal fly ash increased to 180 μm. Here it appeared that cluster formation was even higher than before, suggesting that the as-received coal fly ash was less soluble in the polymer solution than in water. This could have been caused by the weak Van der Waals forces of attraction present between the inorganic fly ash particles. However, for all fly ash samples exposed to acetylene at temperatures between 400°C and 700°C, there was a huge reduction in the particle sizes. Those exposed to acetylene at 500°C recorded the lowest particle size, i.e. 220 nm. For this reason, a particle size distribution, based on the TEM images, was also conducted on these CNFs.

In Figure 6, the materials found in the TEM images of the as-received and acetylene-treated fly ash samples at 500°C were measured. As can be seen, there was a huge reduction in the particle sizes measured by TEM, as compared to when the materials were measured using the particle size analyser (Figure 6). It was noted though that one of the drawbacks of using the particle size analyser was that it did not allow particles to be individually measured. This explains the reduction in size when the data (Figure 6) was compared to the TEM analyses, as particles were individually measured. In the latter case, the average size was found to be 57 and 28 nm for as-received fly ash and CNFs from acetylene-treated coal fly ash, respectively. To confirm these findings, BET was used to study their surface areas (Figure 7). The results showed that the CNFs produced at 500°C displayed the highest surface area (59 m^2^/g). Studies have shown that the lower the particle size, the higher the surface area [12].

### Composition, mineral phase and oxidation state studies

**Figure 8:**
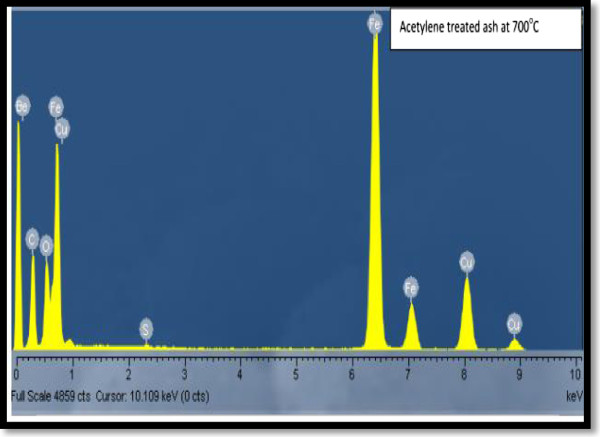
**EDS of CNFs synthesized at 700°C.** Berrylium, carbon, aluminium, silica and iron were the elements identified after synthesis.

**Figure 9:**
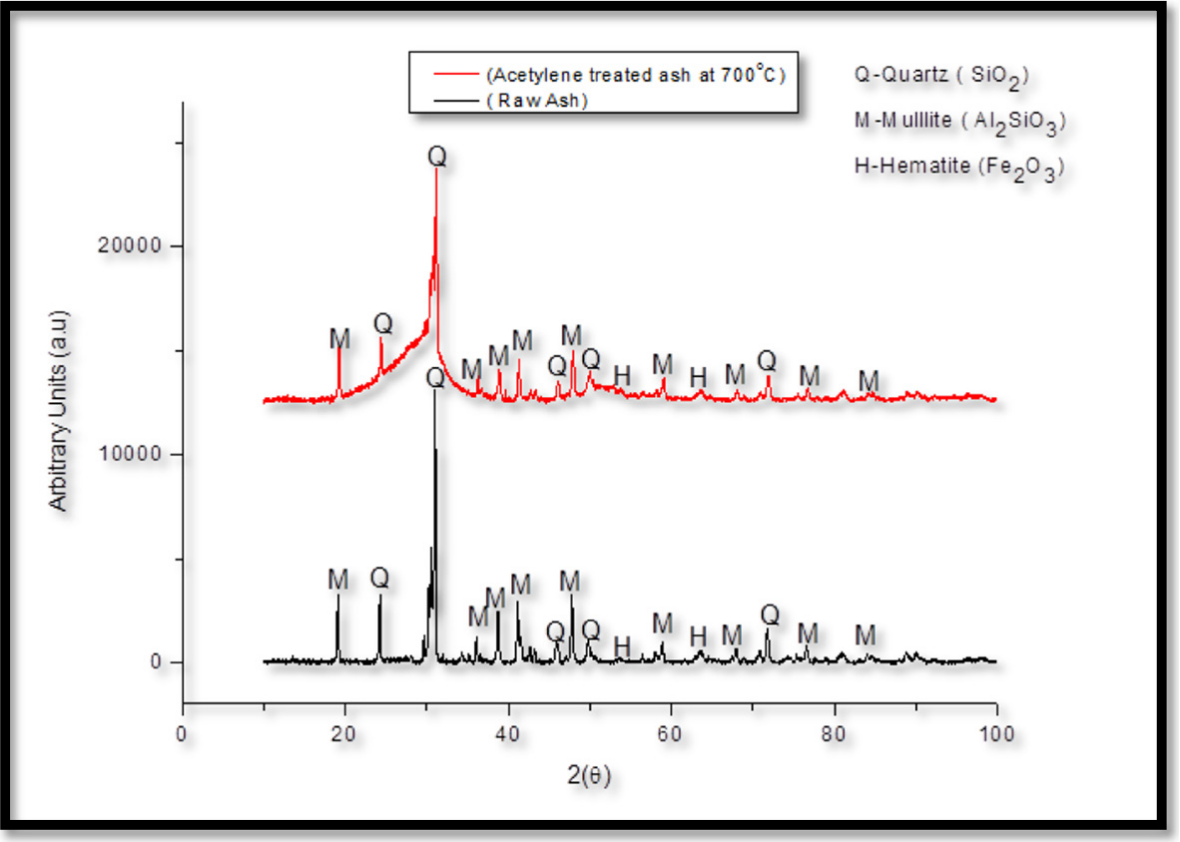
**XRD of as-received coal fly ash and acetylene-treated coal fly ash at 700°C.** As-received coal fly ash contained mullite, quartz and hematite as major phases. After synthesis, peak shifting occurred, the crystallinity changed, and the formation of silicates and Fe phases were more evident.

To confirm which elements were responsible for CNF formation, EDS, XRD and Mössbauer spectroscopy were employed. The catalyst suspected to be responsible for CNF formation was iron. The presence of this element was verified by EDS as displayed in Figure 8. XRD and Mössbauer spectroscopy were then used in an attempt to clarify its connection with CNF formation. As-received and acetylene-treated fly ash samples were then analysed by XRD. These XRD patterns suggested that exposure to heat, acetylene and hydrogen induced significant phase changes to the coal fly ash as displayed in Figure 9.

The major phases in the as-received coal fly ash were quartz (SiO_2_), hematite (α-Fe_2_O_3_) and mullite (3Al_2_O_3_ · 2SiO_2_). After exposure to acetylene, it was noted that peak shifting and broadening had occurred, as was most evident in quartz at 26.5° (2*θ*). This may have been caused by amorphous glassy phases, found in the as-received fly ash, which when exposed to acetylene and hydrogen became more crystalline [12]. The iron content with the presence of silicates also became more apparent after CNF formation. However, the new phase of iron could not be identified by XRD (which is a bulk technique). Previous studies have shown that when iron is in low quantities and high dispersions, some of its phases cannot be identified using XRD [47]. Likewise for iron, it has been shown that in such cases, the exact phase identification by XRD is difficult as it tends to form a large variety of carbides [47]. In one study, cementite (Fe_3_C), which could not be identified by XRD, was observed by Mössbauer spectroscopy during the formation of CNTs over iron catalysts from acetylene decomposition [47]. Hence, ^57^Fe Mössbauer spectroscopy, which is able to identify all forms of iron, was employed in this study. In order to obtain the chemical and structural information of iron-containing materials, three main hyperfine parameters, namely the isomer shift, quadrupole splitting and magnetic splitting, need to be investigated.

**Figure 10:**
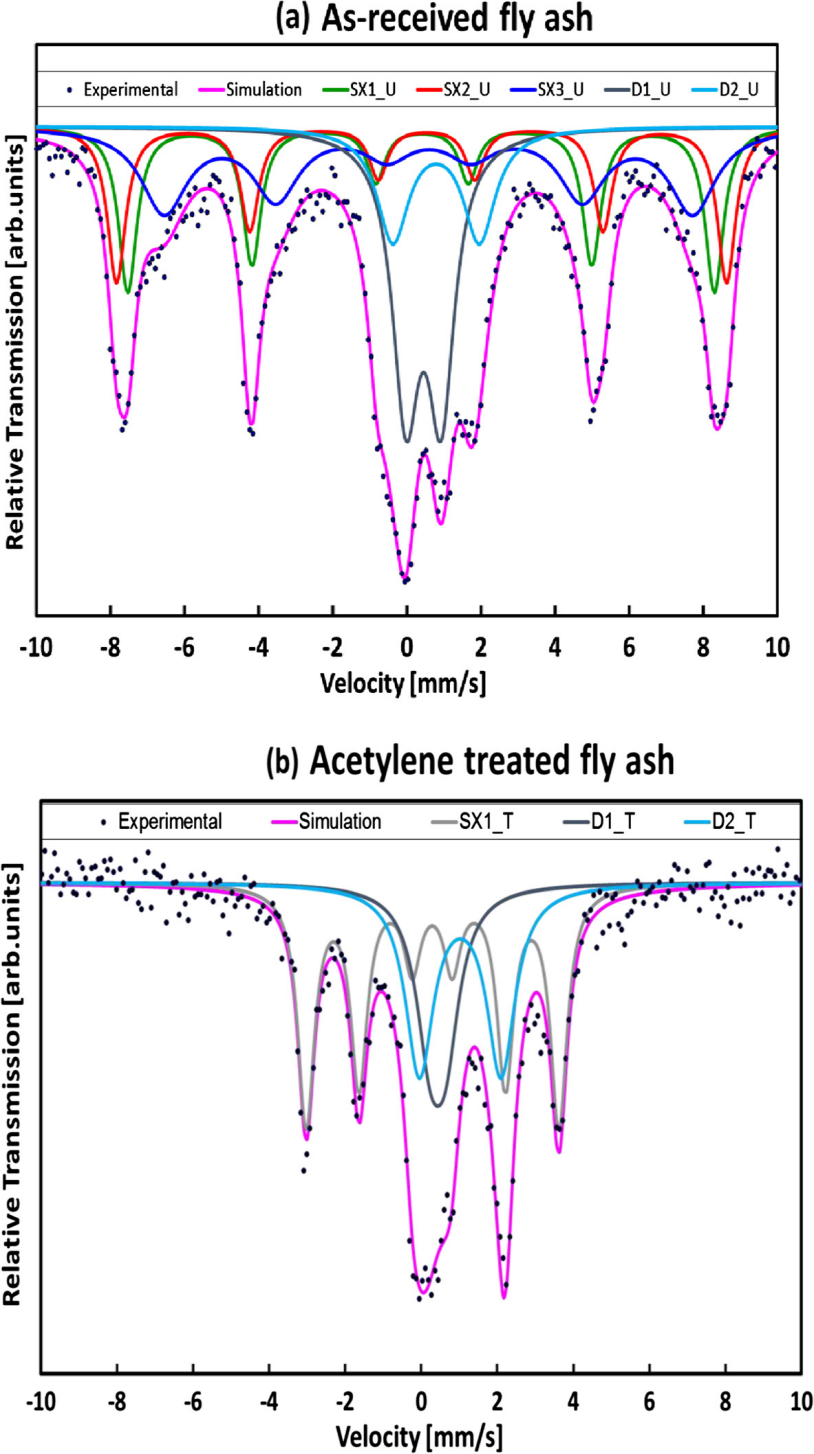
**Room-temperature **^**57**^**Fe Mössbauer spectra for (a) as-received and (b) acetylene-treated coal fly ash sample at 700°C.**

Figure 10a,b shows the fitted spectra obtained for the as-received coal fly ash sample and the sample after being exposed to acetylene. The spectra were characterized by broadened six-line patterns, and the central region was dominated by a distribution of quadrupole split doublets. The magnetic feature for the as-received coal fly ash sample (not subjected to acetylene) was fitted with three sextets (SX1_U, SX2_U and SX3_U), while the spectrum for the acetylene-treated sample was analysed with one sextet (SX1_T). For each spectrum, two doublets were required in the central region to give good fits. Table 2 gives a summary of the hyperfine parameters obtained from the fits to the data for both the as-received and acetylene-treated samples. Isomer shifts and velocities were given relative to the centre of the spectrum of alpha-Fe at room temperature (RT). For the as-received fly ash sample, the hyperfine parameters extracted for SX1_U and SX3_U were as follows: *B*_hf_ = 49.0 T, δ = 0.40 mm/s; Δ*E*_Q_ = −0.02 mm/s and *B*_hf_ = 44.2 T, δ = 0.59 mm/s; Δ*E*_Q_ = −0.01 mm/s. These values corresponded to Fe^3+^ ions on tetrahedral A-sites and Fe^2.5+^-like average signals from octahedral B-sites, respectively, and were identified as magnetite (Fe_3_O_4_). The SX2_U spectral component with hyperfine parameters of *B*_hf_ = 51.6 T, δ = 0.45 mm/s; Δ*E*_Q_ = −0.13 mm/s was attributed to hematite (Fe_2_O_3_). The latter iron oxide was also detected by XRD. For the as-received sample, the hyperfine parameters determined for D1_U and D2_U were δ = 0.45 mm/s; Δ*E*_Q_ = 0.95 mm/s and δ = 0.79 mm/s; Δ*E*_Q_ = 2.33 mm/s characteristic of ferric and ferrous ions, respectively. The quadrupole split doublets were attributed to silicates.

**Table 2:** Room-temperature Mössbauer parameters for as-received and acetylene-treated coal fly ash samples

	**Values**				
As-received	SX1_U	SX2_U	SX3_U	D1_U	D2_U
*B*_hf_ (T)	49.0	51.6	44.2	-	-
δ (mm/s)	0.40	0.45	0.59	0.45	0.79
Δ*E*_Q_ (mm/s)	−0.02	−0.13	−0.01	0.95	2.33
Area (%)	21	18	27	23	11
Treated	SX1_T	D1_T	D2_T		
*B*_hf_ (t)	20.5	-	-		
δ (mm/s)	0.29	0.43	1.02		
Δ*E*_Q_ (mm/s)	−0.003	0.41	2.15		
Area (%)	49	21	30		

The as-received sample showed that the total population of the oxides is 66% and 34% is attributed to silicates. After treatment, a decrease in the area fraction of 17% was observed for the oxides with a corresponding increase in the silicates.

After exposure to acetylene, only one sextet, SX1_T, with a reduced magnetic field was observed in the spectrum with hyperfine parameters of *B*_hf_ = 20.5 T, δ = 0.29 mm/s; Δ*E*_Q_ = −0.003 mm/s which has been identified as nanocrystalline iron carbide (Fe_3_C). The hyperfine parameters of δ = 0.43 mm/s; Δ*E*_Q_ = 0.41 mm/s and δ = 1.02 mm/s; Δ*E*_Q_ = 2.15 mm/s obtained for D1_T and D2_T, respectively, were very similar to those obtained for the as-received sample except for the quadrupole splitting of D1_T which was lower and indicated some structural relaxation.

For the as-received fly ash sample, the total population of the oxides was 66% with the remaining fraction of 34% attributed to silicates. After exposure to acetylene, a decrease in the area fraction of 17% was observed for the oxides with a corresponding increase in the silicates. The abundance of the Fe^2+^ state before treatment was approximately 11% but showed an increase of approximately 19% after acetylene treatment due to the reduced magnetic field.

These results indicate a reduction in the oxidation state of iron (with decreasing oxide content), as a new phase of iron (Fe_3_C) and silica emerged. This suggestion is in agreement with He et al., who have studied Mössbauer spectroscopy of CNT formation from acetylene which reacted over iron-supported zeolite catalysts and who have found that the +3 oxidation state of iron was reduced to +2 by H_2_, which they concluded was the active phase for their synthesis [48]. Dunens et al*.* found that iron also appeared in different forms in their ash and that H_2_ could not reduce these, presumably because of their location in the fly ash particles [36]. Hence, in their study, unlike in this present work, Dunens et al. were required to further impregnate their ash with iron in order for CNT/CNF growth to occur. In a similar manner, Diamond [49], using acid etching techniques, demonstrated that the location of the iron and its morphology greatly differed for every fly ash particle within the sample. This, he suggested, was caused by the inhomogeneous nature of coal.

The magnetic feature for the as-received sample was fitted with three sextets (SX1_U, SX2_U and SX3_U) and the spectrum for the acetylene-treated sample was analysed with one sextet (SX1_T), while the non-magnetic spectral components for both samples were fitted with two quadrupole split doublets.

## Conclusions

CNFs (and a small amount of CNTs) were successfully produced by directly using an as-received South African coal fly ash. The smooth, glassy and inert surfaces of the South African coal fly ash were covered with irregularly shaped CNFs in the presence of acetylene and hydrogen at temperatures as low as 400°C. Laser Raman spectroscopy confirmed the formation of CNFs. TGA showed that there were different forms of carbon present, i.e. graphitic and amorphous. On the other hand EDS, XRD and Mössbauer spectroscopy confirmed that iron, most likely in the form of iron carbide, was directly associated with the formation of CNFs. Therefore, this study has demonstrated the successful synthesis of carbon nanostructured materials from waste South African coal fly ash without chemical pre-treatments (such as the impregnation of other metals) or thermal modifications. Since CNFs may in the future be beneficial for applications such as particulate nanofillers in polymer matrices, this intervention could result in the reduction of environmental pollutants. Concomitantly, this may also bring relief to the financial burden involved in the disposal costs of this and related coal fly ash around the world in the long run.

## Competing interests

The authors declare that they have no competing interests.

## Authors' contributions

NH carried out the experimental work, synthesis, characterization and analysis and wrote the paper. AS participated in the experimental design, carried out the initial baseline work on the study and assisted in constructing the paper. DN and HM ran the Mössbauer, interpreted the results and wrote the section. DB assisted with the analysis of XRD. PF and SD participated in the design and coordination of the study and interpretation of the results. All authors read and approved the final manuscript.

## Authors' information

NH holds a master's degree and is currently a PhD student at the University of the Witwatersrand. AS received his PhD after publishing in high impact factor journals and is now working at the Registrar's office at the University of the Witwatersrand. PF received his PhD from Cambridge University (UK) and is now working as a lecturer at the University of the Witwatersrand. HM holds a PhD and is a lecturer at the School of Physics. DN holds a PhD and is the head of the Mössbauer facility at the School of Physics. DB holds a PhD, is a professor and is the head of the XRD unit at the Wits School of Chemistry. SD holds a PhD and is currently a senior lecturer and research focus area coordinator for CNTs and strong composites in the DST-NRF Centre of Excellence in Strong Materials at the University of the Witwatersrand.
